# MMST: A Multi-Modal Ground-Based Cloud Image Classification Method

**DOI:** 10.3390/s23094222

**Published:** 2023-04-23

**Authors:** Liang Wei, Tingting Zhu, Yiren Guo, Chao Ni

**Affiliations:** College of Mechanical and Electronic Engineering, Nanjing Forestry University, Nanjing 210037, Chinatingtingzhu@njfu.edu.cn (T.Z.);

**Keywords:** ground-based cloud image (GCI) classification, Swin Transformer, global features, feature fusion

## Abstract

In recent years, convolutional neural networks have been in the leading position for ground-based cloud image classification tasks. However, this approach introduces too much inductive bias, fails to perform global modeling, and gradually tends to saturate the performance effect of convolutional neural network models as the amount of data increases. In this paper, we propose a novel method for ground-based cloud image recognition based on the multi-modal Swin Transformer (MMST), which discards the idea of using convolution to extract visual features and mainly consists of an attention mechanism module and linear layers. The Swin Transformer, the visual backbone network of MMST, enables the model to achieve better performance in downstream tasks through pre-trained weights obtained from the large-scale dataset ImageNet and can significantly shorten the transfer learning time. At the same time, the multi-modal information fusion network uses multiple linear layers and a residual structure to thoroughly learn multi-modal features, further improving the model’s performance. MMST is evaluated on the multi-modal ground-based cloud public data set MGCD. Compared with the state-of-art methods, the classification accuracy rate reaches 91.30%, which verifies its validity in ground-based cloud image classification and proves that in ground-based cloud image recognition, models based on the Transformer architecture can also achieve better results.

## 1. Introduction

As a clean new energy source, the advantage of solar power compared to ordinary thermal power systems is that sun light shines on the Earth and can be exploited directly without mining and transportation. Secondly, solar energy has the characteristics of high energy capacity, no pollution, and wide distribution [1]. In the process of large-scale grid-connected photovoltaic power generation, the impact of PV power fluctuations on the grid cannot be ignored. Among the many factors affecting photovoltaic power generation, the most important meteorological factor is solar irradiance, and the condition of cloud cover will directly affect solar irradiance. In cloud observation, there are three main elements: cloud amount, cloud base height, and cloud shape. Among these elements, the cloud shape can instantly reflect the local atmospheric conditions, so the study of the classification of cloud shape is an essential part of cloud observation research.

In the early period, most weather stations relied on manual visual inspection by weather observers for cloud recognition, and the classification effect would vary depending on the observers’ experience. For this reason, researchers have used classical image features to establish traditional machine-learning classification models for ground-based cloud images. However, the cloud recognition effect is not desirable, especially since the recognition rate of clouds in complex backgrounds is difficult to guarantee. Liu et al. [2] used threshold segmentation and morphological methods to detect the edges of clouds and extract structural features from them to build a supervised classifier to classify weather clouds. Heinle et al. [3] extracted cloud color and texture features and obtained a high classification accuracy based on the Leave-One-Out Cross Validation method. Oikonomou et al. [4] adopted Regional Local Binary Pattern (R-LBP) and Four Patch-Local Binary Pattern (FP-LBP) to describe the global and local features of the cloud image, and in the classification stage, they used Support Vector Machine (SVM) and Linear Discriminant Analysis (LDA) classifiers. From the result, the classification results achieved over 90% accuracy with both datasets, much higher than the methods proposed in other papers. Xiao et al. [5] first extracted the original features of ground-based clouds from the perspectives of color, texture, and structure; they obtained more descriptive representation vectors using a dense sampling method and finally encoded the features and put them into the support vector machine for classification.

In recent decades, with the generation of datasets, the improvement of computing capability, and the development of various machine learning algorithms, the utilization of deep learning to deal with specific problems has received growing attention. Zhang et al. [6] proposed a new convolutional neural network (CNN) model, namely, CloudNet. The author proposed that we could achieve better results by combining abundant information from different locations in the ground-based cloud image. However, he did not elaborate on the specific impact of information from different locations on the model. Liu et al. [7] considered each cloud image as a node in the graph; they used GCN to aggregate information from the cloud images themselves and their connected images in a weighted manner to establish strong and weak connections between different classes of cloud images and mine the inherent structural information of clouds. Although Transformer [8] architecture has become a fundamental model in the natural language processing field, its application in computer vision still needs to be improved. In computer vision, attention mechanisms are used in conjunction with or to replace certain parts of convolutional networks. Therefore, inspired by the Transformer, Alexey et al. [9] proposed the Vision Transformer (Vit), which mainly divides the image into multiple blocks, called Patches, and then embeds them into the linear layer of the Transformer. Li et al. [10] applied the Swin Transformer to cloud image classification but still added a convolutional layer to the overall model to extract local information without using meteorological multi-modal information to assist in the classification. The formation of clouds is affected by many natural factors [11]. Therefore, utilizing this multi-modal information is significant for comprehensively characterizing clouds. Liu et al. [12] proposed a new ground-based cloud classification method, namely, the multi-level modality fusion model (HMF), which fuses deep multi-modal features and deep visual features at different levels, namely, low-level fusion and high-level fusion. High-level fusion combines the output of low-level fusion with visual features and multi-modal features.

Therefore, this paper proposes a ground-based cloud image classification model based on the multi-modal Swin Transformer (MMST). Without using the convolution module, the visual backbone network can perform well, integrating multi-modal information to enhance the model’s representation ability further. The approach outperforms the currently available methods in the publicly available multi-modal base cloud image dataset MGCD [12], demonstrating its feasibility.

The main contributions of this paper are as follows:

1. This paper proposes a novel method based on the Swin Transformer. The model fully relies on the attention mechanism and the linear layer to learn the features of cloud images and multi-modal information. This method solves the shortcomings of the traditional CNN model, namely, that it cannot conduct global modeling, and the performance ceiling of the model is restricted by too much inductive bias.
2. We address the deficiency of learning only the modeling of images in the cloud classification task. Residual blocks are added to the linear layer to learn more complex feature representations of meteorological multi-modal information.
3. An experimental evaluation is carried out on the multi-modal base cloud image dataset MGCD, showing that the method proposed in this paper has better classification results.

## 2. Methods

### 2.1. Overview of the Classification Process

**Figure 1:**
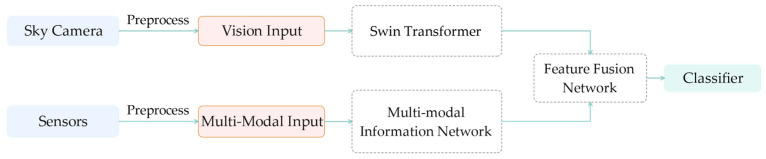
The classification process of ground-based cloud images.

This paper proposes the MMST model for the ground-based cloud image classification task. First, the original ground-based cloud image (the image size is 1024 × 1024) is taken by the all-sky camera, and four kinds of original meteorological information (temperature, humidity, pressure, and wind speed) are collected by the sensor. The original ground-based cloud image and original meteorological information are then pre-processed, and the processed ground-based cloud image and meteorological information are input, respectively, into the visual backbone network Swin Transformer and Multi-modal Information Network to, respectively, obtain the Vision Feature and Multi-Modal Feature. Finally, they are sent to the Feature Fusion Network, the Concat operation is conducted, and the classification result is output by the Linear layer. The overall classification process is shown in Figure 1.

### 2.2. Introduction of the Proposed Method

**Figure 2:**
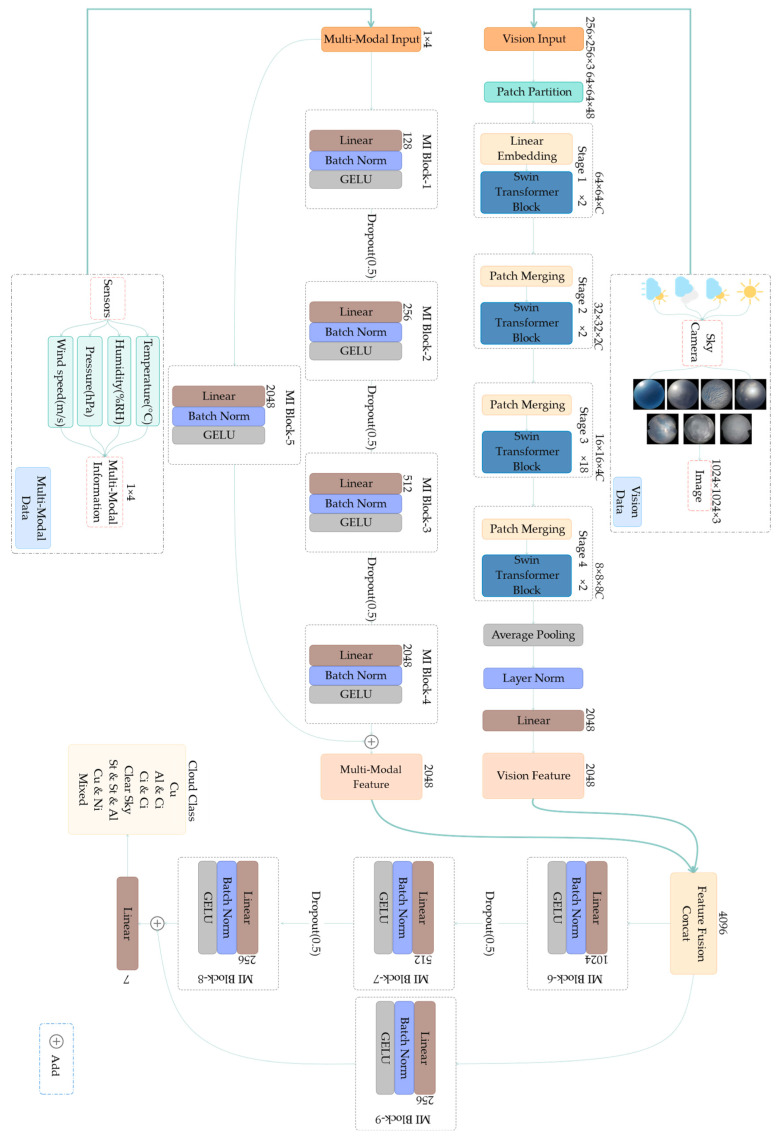
The structure of the proposed classification network (MMST).

Figure 2 shows the overall structure of MMST. The visual backbone network Swin Transformer learns the feature relationship between image patch sequences by calculating the attention that the Patch can achieve for the purpose of global modeling. Shifting the windows can achieve the effect of fusing local information by interacting with the windows, similar to the convolution in CNN. With patch merging, the feature map size is downsized to achieve cascading multi-scale features and extract higher-level information, such as pooling in CNN. Moreover, the Multi-modal Information Network and Feature Fusion Network consist of blocks composed of linear layers to learn better feature representation using the residual structure, and the presence of the residual unit also ensures the stability of the gradients in model training.

### 2.3. Visual Backbone Network

#### 2.3.1. Swin Transformer V2

In the MMST model, Swin Transformer V2 [13] is used as the vision backbone network. Swin Transformer V2 is an improved version of the Swin Transformer [14] (i.e., the V1 version), which is also a model based on the Vit architecture. Due to the similarity of the main structure, in this article, the V1 and V2 versions of the Swin Transformer are not distinguished by name. The improvements proposed by the V2 version relative to the V1 version are roughly divided into two parts as follows:

(1) Improvements to self-attention

**Figure 3:**
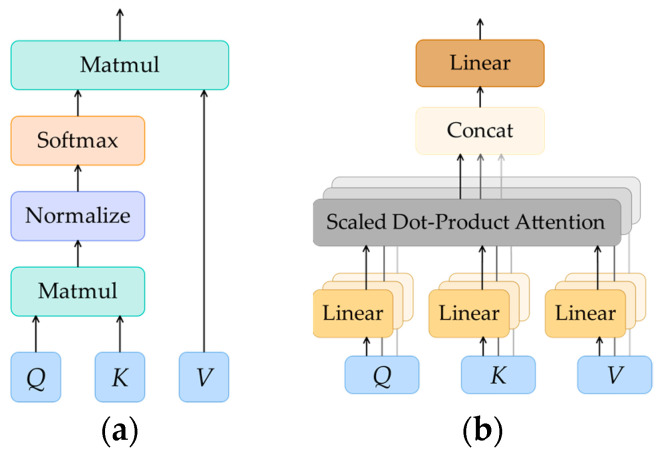
(**a**) The self-attention and (**b**) the multi-head self-attention.

For the original Vit model, the two-dimensional (2D) image is first segmented, and the image of $X_{p}\in R^{H\times W\times C}$ is divided into several non-overlapping patches, namely, $X_{p}\in R^{P\times P\times C}$, where H×W is the size of the original image, C is the number of channels in the image, and P×P is the size of each patch. Since the spatial position of each patch has some influence on the later classification, position encoding is added to the vector of the patch projections to preserve their spatial information. The core components of the Vit encoder are a multi-head self-attention (MSA) module and a feed-forward multilayer perceptron (MLP) [8]. Figure 3 illustrates the structure of the MSA module, which is composed of multiple self-attention. The structure of self-attention is shown in Figure 3. The input vector is transformed by three trainable matrices to obtain three different homologous matrices: query matrix, *Q*; key matrix, *K*; and value matrix, *V*. The weight of each element (i.e., the importance of each element to the context) is obtained by computing the dot product of the query matrix and the key matrix and then applying Softmax to scale the weight values to the interval of (0, 1). The transformed weight is multiplied by the value matrix to obtain the element value carrying the global importance information, which is different from local modeling in CNN [15]. The calculation of self-attention is as follows:

(1) $$Attention\left(Q,K,V\right)=Softmax\left(\frac{QK^{T}}{\sqrt{d_{k}}}\right)\cdot V,$$

where $\sqrt{d_{k}}$ is the square root of the key matrix dimension, the purpose is to make the weight distribution smoother and more reasonable, and also to make the gradient more stable. MSA divides the input into n parts, i.e., n-head self-attention, performs the attention calculation of Equation (2) on the n parts of the input, and obtains n attention output results. Finally, the n output is concatenated and restored into the original dimension through the linear layer. The MSA calculation process is shown as follows:

(2) $${\mathrm{head}}_{i}=Attention\left(QW_{i}^{Q},KW_{i}^{K},VW_{i}^{V}\right),$$

(3) $$MSA\left(Q,K,V\right)=Concat\left({\mathrm{head}}_{1},\;\ldots ,{\mathrm{head}}_{i}\right)W^{0},$$

where i is the number of input vectors divided, ${\mathrm{head}}_{i}$ is the attention output of the ith head, and the $W_{i}^{Q},W_{i}^{K},W_{i}^{V}$, and $W^{0}$ are all learnable parameter matrices.

**Figure 4:**
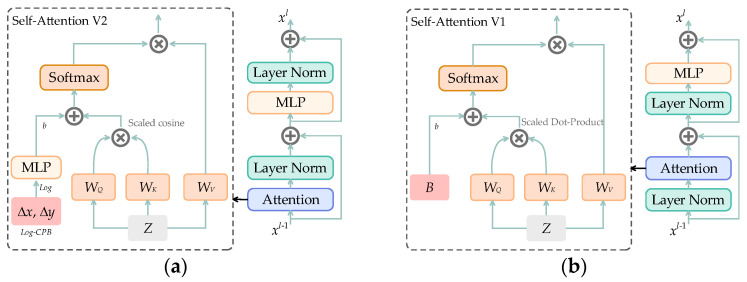
(**a**) The self-attention V2 and (**b**) the self-attention V1.

Scaled cosine attention [13] is used in Swin Transformer V2 instead of Scaled Dot-Product attention, as shown in Figure 4. A comparison of self-attention V1 and V2 is shown in Figure 4, where Figure 4 shows the improved structure of self-attention in V2, and Figure 4 shows the original structure. Scaled cosine attention is proposed to solve a problem that arises in the res-post-norm configuration, namely, that the learned attention maps of some blocks and heads are frequently dominated by a few pixel pairs. Scaled cosine attention can compare the similarity of each element in two vectors, and its calculation is as follows:

(4) $$Scaled\;cosine\;attention\left(q_{i},k_{j}\right)=cos\left(q_{i},k_{j}\right)/\tau +B_{ij},$$

where i and j are different pixel coordinate indices, q and k are query and key vectors, respectively, $B_{ij}$ is the relative position bias of pixels at i and j coordinates, and τ is a globally shared learnable scalar. The cosine function is naturally normalized and thus can have milder attention values.

(2) Improvements to shifted windows multi-head self-attention

**Figure 5:**
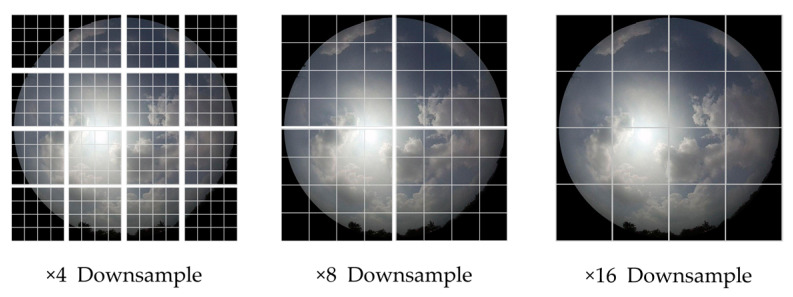
Hierarchical feature maps in Swin Transformer.

The Swin Transformer is proposed to build hierarchical feature maps by merging image patches [16], as shown in Figure 5. Compared with the method of keeping the feature map size invariant in Vit, hierarchical feature maps not only use multi-scale features for modeling, but they can also greatly reduce the complexity of self-attention operations [14]. The model based on Transformer architecture itself cannot implicitly learn the position information of the sequence, and in the process of dividing the picture into multiple patches, the border between the patches has a specific meaning; therefore, the Swin Transformer adds relative position encoding (RPE) to each patch [17], which can compensate for the relative position information between the two elements that is lost when computing self-attention with absolute position encoding. Therefore, the self-attention calculation formula using relative position encoding is as follows:

(5) $$Attention-RPE\left(Q,K,V\right)=Softmax\left(\frac{QK^{T}}{\sqrt{d_{k}}}+B\right)\cdot V,$$

where $B\in R^{M^{2}\times M^{2}}$, the value in *B* comes from a smaller-sized bias matrix $\hat{B}\in R^{\left(2M-1\right)\times \left(2M-1\right)}$, $M^{2}$ is the number of patches in a window, and Q,K,V are the query matrix, key matrix, and value matrix, respectively.

**Figure 6:**
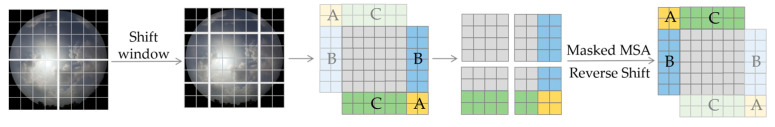
Illustration of the self-attention in shifted window partitioning.

The Swin Transformer uses the concept of Windows Multi-head Self-Attention (WMSA) to partition the image into multiple non-intersecting windows, and Multi-head Self-Attention is only computed in each individual Window. However, in Vit, Multi-head Self-Attention is directly performed on the global Window. The purpose is to reduce the amount of calculation, but this occurs at the expense of information transmission between different Windows. Therefore, in this paper, we propose using Shifted Windows Multi-head Self-Attention (SW-MSA) to address the shortcomings of WMSA. Through this method, information can be transmitted in adjacent windows. The block based on Windows Multi-head Self-Attention (W-MSA) and the block based on Shifted Windows Multi-head Self-Attention (SW-MSA) constitute the core components of the Swin Transformer. W-MSA refers to the mutual calculation of self-attention between patches in a Window with a specified size. Compared with MSA in Vit, W-MSA can effectively save computing resources and improve model computing efficiency. Equations (6) and (7) represent the computational complexity of MSA and W-MSA, respectively:

(6) $$\Omega \left(MSA\right)=4hwC^{2}+2{\left(hw\right)}^{2}C,$$

(7) $$\Omega \left(W-MSA\right)=4hwC^{2}+2M^{2}hwC,$$

where h, w, and C represent the height, width, and channel of an image, respectively, M denotes that each window contains M×M patches. The former is quadratic to patch number h×w, and the latter is linear when M is fixed. The difference between the two computational complexities increases as the input image size increases. SW-MSA implements communication between windows through the Shift window, Masked MSA, and Reverse shift, as detailed in Figure 6.

To improve the stability of the model and the performance loss caused by the SW-MSA process, Swin Transformer V2 uses log-spaced continuous position bias to solve the above problem. The introduction of a log-spaced continuous position bias approach guarantees that the relative position bias can be smoothly transferred across window resolutions. The continuous position bias method uses a small meta-network (e.g., an MLP network) in relative coordinates to optimize the bias parameters, which is different than directly optimizing the parameters in a traditional network:

(8) $$B\left(\Delta x,\Delta y\right)=G\left(\Delta x,\Delta y\right),$$

where G is a small network that can be designed artificially. Then, to alleviate the computational problem of needing to calculate relative position coordinates when converting between windows and the problem of inconsistent relative position coordinate ranges for windows of different sizes,

(9) $$\begin{array}{c} \hat{\Delta x}=sign\left(x\right)\cdot log\left(1+\left|\Delta x\right|\right),\\ \hat{\Delta y}=sign\left(y\right)\cdot \log\left(1+\left|\Delta y\right|\right), \end{array}$$

where Δx, Δy and $\hat{\Delta x}$, $\hat{\Delta y}$ are the linear-scaled and log-spaced coordinates, respectively.

#### 2.3.2. Feature Map Visualization

**Figure 7:**
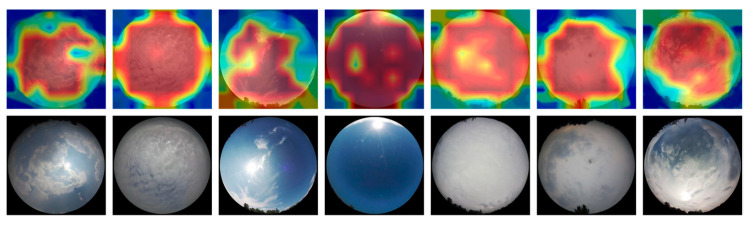
Grad-CAM++ visualization results in the MGCD dataset.

To illustrate the feature extraction ability of the proposed method more intuitively, we used the Gradient-weighted Class Activation Mapping++ (Grad-CAM++) [18] method for feature visualization. The method shows the crucial regions of the image predicted by generating a rough attention map from the chosen layer of the model. For our work, we used the last norm layer of the last block of the Swin Transformer as the chosen layer. The redder the color of the attention map, the higher the importance of the corresponding region of the image. The visualization results are shown in Figure 7. The proposed method can focus on the critical parts of the cloud image, and for images with fewer clouds, the model expands the area of focus (e.g., the third image from left to right). The model will focus on the overall features of images with dense clouds and no apparent features.

### 2.4. Multi-Modal Information Network and Feature Fusion Network

In the Multi-modal Information Network and the Feature Fusion Network, we utilize the Multi Information Block (MI Block) as the main component. The MI Block consists of Linear, Batch Norm, and GELU, and the Dropout layer is used between MI Blocks to prevent overfitting; the default setting of the Dropout ratio is 0.5. Regarding the choice of activation function, we use the Gaussian Error Linear Unit (GELU) [19]. In the modeling process of neural networks, the fundamental property of the model is non-linearity. At the same time, it is necessary to include the stochastic regular for the model generalization ability. The non-linear activation and the stochastic regular determine the model’s input. In the activation, the *GELU* introduces the idea of the stochastic regular, a probabilistic description of the input of the neuron, which is calculated as follows:

(10) $$GELU\left(x\right)=0.5x\left(1+tanh(\sqrt{2/\pi }(x+0.044715x^{3}))\right),$$

In addition to the regularization operations employed above, MMST uses residual units between certain MI Blocks. This idea is borrowed from the Deep crossing model [20], where the use of residual units in the designed network structure is likely to perform regularization, ensuring the stability of the model implicitly.

### 2.5. Implementation Details

We downscaled the ground-based cloud image from 1024 × 1024 to 256 × 256 and imported it into MMST at this size. Then, to perform data augmentation on the image, we used random horizontal and vertical flips with a probability of 50%. Subsequently, each image was normalized according to the mean and variance of ImageNet. On the other hand, to ensure data matching, we normalized the values of multi-modal information with normal distribution.

The experimental platform comprised a server containing an Intel(R) Core(TM) i9-9900 K 3.60 GHz CPU, an NVIDIA GTX 2080 Ti, and a memory of 32 GB. The operating system was Windows 10 Professional Edition. The software environment was Python3.10 in Pytorch 1.11.

In terms of models, the main network was initialized by the pre-trained Swin Transformer V2 on the ImageNet dataset, and we fine-tuned it on MGCD. For the parameters of the linear layer in the Multi-modal Information Network and Feature Fusion Network, we used Kaiming Initialization [21], which can solve the problem that Xavier Initialization [22] is only applicable to linear activation functions and guarantees the stability of the gradient to a certain extent. Adding Batch Norm after each linear layer and using Dropout between MI Blocks can effectively prevent the model from overfitting. During the training phase, we used the NAdam optimizer to update the parameters of the network. NAdam adds the accumulation of Nesterov momentum on the basis of Adam. NAdam has more substantial constraints on the learning rate and has a more direct impact on the updating of the gradient. The initial learning rate was set to 5 × 10^−6^. Limited by the experimental equipment, the total number of training iterations was set to 30 and the batch size was set to 16. The computation complexity of MMST was 15.246 GFLOPs and the number of MMST’s parameters was about 74.104 M. The total training time was about 2.5 h. The loss function was Cross Entropy Loss, which is calculated as follows:

(11) $$Loss=-\sum_{c=1}^{c}w_{c}\log\frac{\exp(x_{n,c})}{\sum_{c=1}^{c}\exp x_{n,c}}y_{n,c},$$

where x is the input, y is the target, w is the weight, and C is the number of classes. *Accuracy* is calculated as follows:

(12) $$\mathrm{Accuracy}=\frac{n_{\mathrm{correct}}}{n_{\mathrm{total}}},$$

where $n_{\mathrm{correct}}$ is the number of correctly classified samples, and $n_{\mathrm{total}}$ is the number of all samples in the test dataset.

## 3. Data Collection

**Figure 8:**
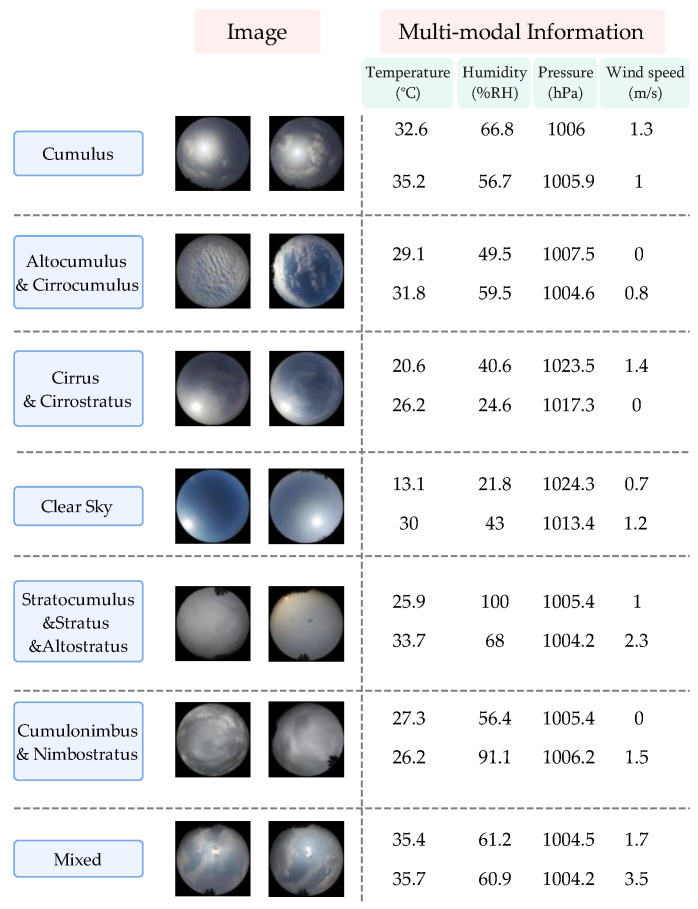
Some samples from the MGCD dataset.

The Multi-modal Ground-based Cloud Image Dataset (MGCD) combines meteorological cloud images and corresponding multi-modal information. MGCD contains 8000 ground-based cloud samples, and each sample includes a cloud image with a resolution of 1024 × 1024 and a set of multi-modal information. The cloud image is collected by a fisheye lens sky camera, which can provide observations of a wide range of sky conditions at 180° horizontal and vertical angles. Multi-modal information includes temperature (°C), humidity (%RH), pressure (hPa), and wind speed (m/s). According to the genus-based classification recommendations of the World Meteorological Organization (WMO), the collected ground-based cloud images are divided into seven categories: (1) Cumulus, denoted as Cu; (2) Altocumulus and Cirrocumulus, denoted as Al-Ci; (3) Cirrus and Cirrostratus, denoted as Ci-Ci; (4) Clear sky, denoted as Cs; (5) Stratocumulus and Stratus and Altostratus, denoted as St-St-Al; (6) cumulonimbus and nimbostratus, denoted as Cu-Ni; and (7) mixed cloud, denoted as Mc. Note that mixed clouds are meteorological clouds in which the sky is usually covered by no less than two types of clouds, and clear skies are cloud images with no more than 10% cloud volume. Figure 8 shows an example of each type of cloud and the corresponding multi-modal information in the MGCD.

## 4. Results

In this section, we compared the classification performance of variants of MMST, hand-crafted, and learning-based methods, and other classical classification methods on MGCD, verifying the effectiveness of the proposed MMST classification.

### 4.1. Comparison with Variants of MMST

**Figure 9:**
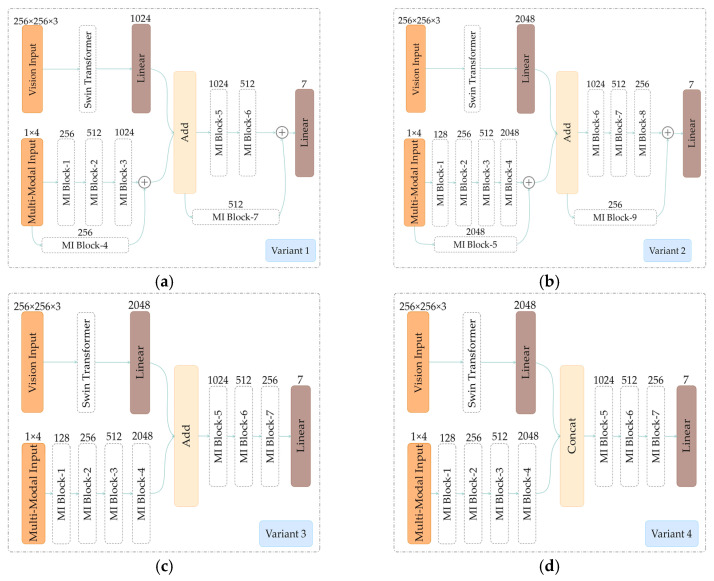
The four different variants of MMST. (**a**) Variant 1. (**b**) Variant 2. (**c**) Variant 3. (**d**) Variant 4.

The advantage of the proposed MMST model is that it can combine ground-based cloud images and corresponding meteorological information to improve the classification accuracy further. To demonstrate their effectiveness on the MGCD dataset, we listed several variants of MMST. The structures of variant 1 through variant 4 are shown in Figure 9. 

**Variant 1.** Variant 1 is a version of MMST, called MMST-small, with a smaller number of parameters (MMST is also called MMST-base). Compared with the C of the Swin Transformer Block in MMST-base, which is 128, MMST-small sets C to 96, the output layer uses fewer neurons (1024), and the feature fusion method uses **Add**. To further reduce the number of MMST parameters, we reduced the number of MI Blocks from 9 to 7.

**Variant 2.** The structure of variant 2 is basically the same as that of MMST-base. The difference is that the **Concat** operation of Feature Fusion is changed to the **Add** operation to verify that for MMST, **Add** or **Concat** is more effective for feature fusion.

**Variant 3.** Variant 3 is based on Variant 2, using **Add** to fuse ground-based cloud images and multi-modal meteorological features. The difference is that the MI Block residual connection is removed, and the rest of the structure remains unchanged to verify the impact of the residual connection on deep fusion.

**Variant 4.** Variant 4 is based on MMST-base and uses **Concat** to fuse ground-based cloud images and multi-modal meteorological features. The difference is that the MI Block residual connection is removed, and the rest of the structure remains unchanged to verify the impact of the residual connection on deep fusion.

**Figure 10:**
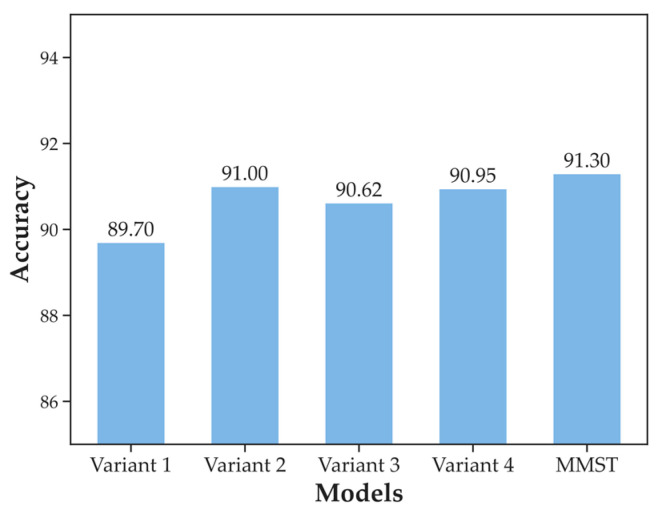
Comparison of accuracy of variants of MMST classification results.

Figure 10 gives the classification accuracy of MMST and its different variants on the MGCD dataset with both inputs of the ground-based cloud image and multi-modal information. The MMST model achieved the highest accuracy at 91.30%.

**Figure 11:**
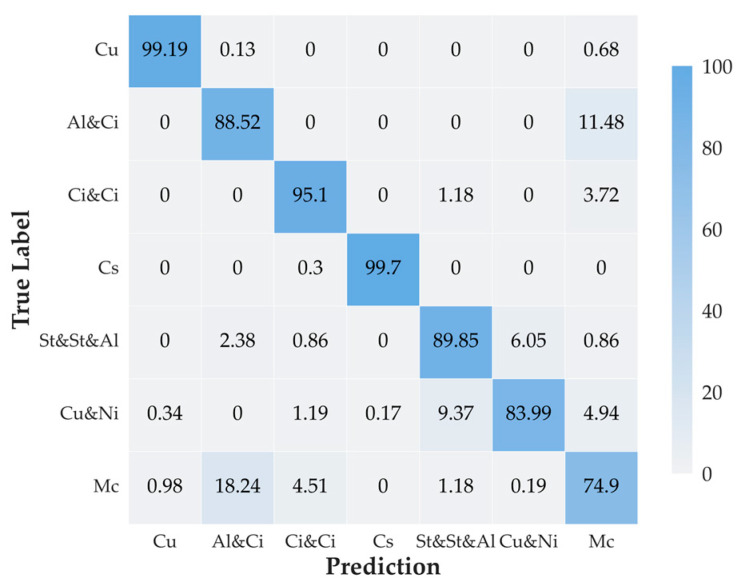
Confusion matrix for MMST classification results.

To further analyze the performance of the proposed MMST model, its confusion matrix is shown in Figure 11, and the Recalls and F1-scores of the proposed MMST for different classes of clouds are listed in Table 1.

### 4.2. Comparison with Hand-Crafted Methods

In this section, we used local binary patterns (LBP) [23] and completed LBP (CLBP) [24], bag-of-visual-words (BoVW) [25], and pyramid BoVW (PBoVW) [26] to conduct experiments on the MGCD dataset to explore the performance of the hand-crafted methods. In the experiments of this subsection, the values of (P, R) of LBP were set to (8, 1), (16, 2), and (24, 3), respectively. Table 2 illustrates the classification results of these methods on the MGCD dataset. The proposed MMST achieved the greatest performance with vision inputs or vision+MI inputs among these classification models.

### 4.3. Comparison with Other Deep Learning Methods

**Figure 12:**
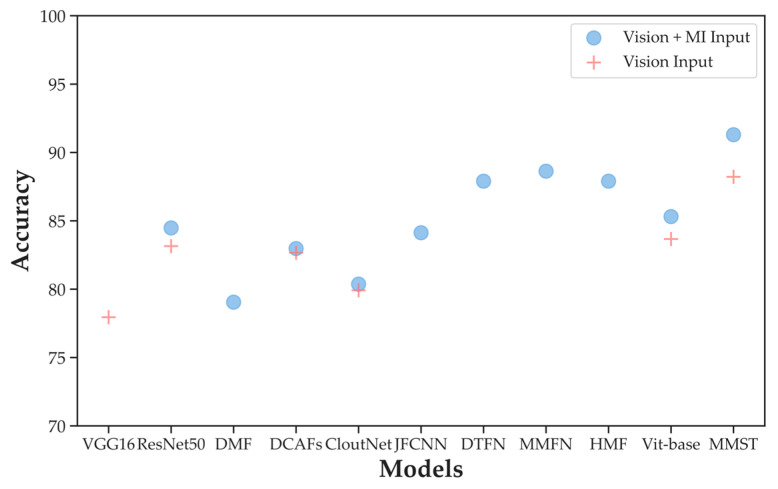
Comparison with other classification methods in two input cases.

Since MMST is a neural network model trained with an end-to-end architecture, we compare MMST with other deep-learning methods (e.g., VGG16 [27], ResNet50 [28], DMF [29], DCAFs [30], CloudNet [6], JFCNN [31], DTFN [32], MMFN [12], HMF [33], Vit-base [9]) in both input cases. The choice of deep learning architecture will also affect the results [34], so the models for comparative experiments in this section include CNN and Transformer architectures. The results are shown in Figure 12.

## 5. Discussion

### 5.1. Analyses of the Experiments with Variants of MMST

Comparing the performance of the proposed MMST and variant 2, as shown in Figure 10, it shows that using the Swin Transformer as the visual backbone network and using the residual structure to fuse multi-modal features have positive effects. The difference between MMST and variant 2 is whether the feature fusion layer uses **Add** or **Concat**. The experimental results showed that using the **Concat** method can retain information to the greatest extent, but this is at the cost of increasing the number of calculations, while using the **Add** method is a special form of **Concat**. That is, the dimension of the image description itself does not increase, but the increasing amount of information under each dimension reduces the computational effort; however, the disadvantage is that the feature information will be lost. For the MGCD dataset, it is more advantageous to use **Concat** to fuse the modal information. Secondly, variant 1 has fewer parameters, which means a faster calculation speed. Compared with MMST and other variants, variant 1 has the lowest classification accuracy. 

Finally, comparing variants 2, 3, and 4 and the proposed MMST, we discovered that one of the differences is in the use of the residual structure. As seen from the results, we found that the model using the residual structure was more effective than the model without the residual structure. Specifically, variant 2 classification accuracy was 0.38% higher than that of variant 3, and MMST was 0.35% higher than that of variant 4. In terms of the information fusion method, variant 2 and variant 3 used the **Add** method, and variant 4 and MMST used the **Concat** method. The results showed that for the same architecture, the model performance was improved by simply replacing **Add** with **Concat**, with MMST achieving an accuracy 0.30% higher than that of variant 2, and variant 4 achieving an accuracy 0.33% higher than that of variant 3.

Combining Figure 11 and Table 1, we can see that the accuracy of Cumulus (Cu), Cirrus and Cirrostratus (Ci-Ci), and Clear sky (Cs) reached 99.19%, 95.10%, and 99.7%, respectively. It is clear from combining the datasets that these three categories of cloud images had clear contours and distinct features relative to other categories. Recall and F1-score both reach above 95%. In contrast, Stratocumulus and Stratus and Altostratus (St-St-Al) and Cumulonimbus and Nimbostratus (Cu-Ni) had no obvious cloud features, and the overall color of the cloud image was grayish, which made it more challenging to distinguish the clouds from the sky. The worst classification effect of cloud type was of mixed cloud (Mc), with Precision, Recall, and F1-score results of only 79.32%, 75.28%, and 77.51%, respectively. The existence of at least two types of clouds within the image increased the difficulty of model learning. Observing the cloud map, we could see that the distribution and shape of mixed cloud (Mc) and Altocumulus and Cirrostratus (Al-Ci) clouds are more similar, so the model can easily misclassify them as Altocumulus and Cirrostratus (Al-Ci).

### 5.2. Analyses of the Experiments with Hand-Crafted Methods

By comparing the classification effects of Vision Input and Vision + MI Input, as shown in Table 2, the BoVW and PBoVW models based on the bag-of-words model were limited by the size of the lexicon as well as the dimensionality; even though the features of the ground-based cloud map were extracted, they could not describe the complete information of the cloud image well. After adding multi-modal information, it had richer features, so the accuracy increased to 67.20%. PBoVW was similar to BoVW, except that it incorporated a pyramidal hierarchical feature extraction technique, which was even less effective than BoVW. LBP had the highest accuracy of only 50.53% (with the combination of parameters (P, R) of (24, 3)) because of its features, such as rotation invariance and grayscale invariance, but it was mainly used to describe local texture features. According to the experimental results, CLBP had a considerable improvement compared to LBP, and the classification accuracy reached 69.68% under the setting of P = 24, R = 3 with the image and multi-modal information jointly input into CBLP, which was 2.53% higher than PBoVW (Vision + MI Input) and 19.15% higher than ${\mathrm{LBP}}_{24,3}^{riu2}$ (Vision + MI Input); however, the accuracy of this classification method never exceeded 70%. It is evident that the classification method based on manual feature extraction has certain constraints, which are not only limited by the difficulty of designing features but also depend on the effectiveness of the extracted features. Compared with the MMST model proposed in this paper, even the best performing ${\mathrm{CLBP}}_{24,3}^{riu2}$ was 19.04% and 21.62% lower than MMST in Vision Input and Vision + MI Input cases, respectively, which shows that the performance ceiling of the machine learning-based classification algorithm is far from the ceiling of the deep learning model.

### 5.3. Analyses of the Experiments with Other Deep Learning Methods

From Figure 12, we can outline the points as follows. Firstly, multi-modal features are complementary to visual features, and their combination can improve the performance of single visual features as input. Secondly, the CNN-based methods, such as CloudNet, JFCNN, DTFN, and so on, are much better than the hand-crafted methods, and the classification accuracies are all higher than 75%. This is attributed to the highly nonlinear transformation nature of CNNs, which enables them to extract effective features from highly complex cloud data. Thirdly, although better than the classical CNN model ResNet50 (0.83% higher), the Vit-base model is less effective than the one designed for MGCD. Owing to the lack of local modeling capability, the results are not as good as the well-designed CNN models for MGCD, such as DTFN, MMFN, and HMF. Fourthly, the proposed MMST improves the accuracy from 90% to 91.30%. Moreover, it works well in both Vision Input and Vision + MI Input, and the classification accuracy in the case of Vision Input is 88.22%, which verifies the effectiveness of the MMST model. 

In addition to the classification-model-based supervised learning discussed in this section, in the development process of deep learning, relevant researchers also proposed multi-task learning and weak supervised learning. Multi-task learning is usually used to deal with different tasks through multiple different models and different loss functions for input data. However, for ground-based cloud image classification tasks, rich feature information can already be obtained from cloud images and multimodal data, so obtaining auxiliary information from multi-task learning is very limited. On the other hand, weak supervised learning only labels part of the data, and the rest of the data depends on the model for reasoning. However, the annotation of ground-based cloud maps requires a lot of professional knowledge and the complexity of cloud movement increases the difficulty of weak supervised learning.

## 6. Conclusions

In this paper, we introduced a novel multi-modal ground-based cloud map recognition method called MMST. The proposed MMST uses only the attention mechanism and linear layers to extract cloud maps and multi-modal features. Since the Transformer architecture lacks CNN-like prior knowledge, the improved Swin Transformer was used as the visual backbone network, where W-MSA and SW-MSA replace the local modeling in the CNN and use improved Scaled cosine attention, residual post normalization, and log-spaced continuous position bias to further promote the model representation. In addition, we incorporated the residual structure into the visual and multi-modal information fusion to ensure the stability of the training process and the adequacy of information fusion. We performed validation on the MGCD and the results show that the proposed MMST is comparable to state-of-the-art methods.

In future work, the following four processes can be considered to improve the proposed model:

1. Collect a ground-based cloud image dataset with a considerably larger amount of data.
2. Obtain more multi-modal information combined with image information to improve classification accuracy.
3. Improve the image coding methods and local modeling capabilities to enable the model of the Transformer architecture to gradually surpass or even replace CNN in the visual field. At the same time, the ability of the model to distinguish mixed cloud layers should be improved.
4. Scaling images before entering the network results in a loss of information. In theory, the Swin Transformer can process input data of any length (that is, images of any size). Future work can be directed toward processing high-resolution ground-based cloud images.

## Figures and Tables

**Table 1:** Evaluation metrics for MMST classification results.

Class	Precision (%)	Recall (%)	F1-Score (%)
Cu	99.13	99.62	99.03
Al-Ci	73.95	89.23	80.27
Ci-Ci	95.78	95.49	95.91
Cs	100	100	100
St-St-Al	86.17	90.55	88.17
Cu-Ni	95.49	84.13	89.04
Mc	79.32	75.28	77.51

**Table 2:** Traditional machine learning classification results.

Class	Vision Input				Vision + MI Input			
	Accuracy (%)	Precision (%)	Recall (%)	F1-Score (%)	Accuracy (%)	Precision (%)	Recall (%)	F1-Score (%)
BoVW	66.15	62.80	66.95	63.94	67.20	66.19	67.91	66.60
PBoVW	66.13	63.53	65.51	64.54	67.15	67.00	65.85	65.23
${\mathrm{LBP}}_{8,1}^{riu2}$	45.38	44.33	45.94	44.99	45.25	46.22	45.07	45.65
${\mathrm{LBP}}_{16,2}^{riu2}$	49.00	49.27	51.34	49.85	47.25	49.53	51.58	50.13
${\mathrm{LBP}}_{24,3}^{riu2}$	50.20	49.55	52.96	50.08	50.53	46.94	49.31	47.11
${\mathrm{CLBP}}_{8,1}^{riu2}$	65.10	64.45	65.39	64.32	65.40	65.12	65.57	65.07
${\mathrm{CLBP}}_{16,2}^{riu2}$	68.20	67.88	67.47	67.78	68.48	69.19	68.18	68.68
${\mathrm{CLBP}}_{24,3}^{riu2}$	69.18	70.71	66.20	68.73	69.68	69.92	71.67	70.50
MMST	**88.22**	**86.87**	**87.48**	**86.79**	**91.30**	**89.86**	**90.17**	**89.17**

## Data Availability

The MGCD dataset is available at https://github.com/shuangliutjnu/Multimodal-Ground-based-Cloud-Database, 17 February 2023.
